# Structured prompting as reusable clinical tools

**DOI:** 10.1016/j.ero.2026.100230

**Published:** 2026-08-21

**Authors:** Dina Husum, Thomas Hügle

**Affiliations:** 1 Danish Centre for Expertise in Rheumatology (CeViG), Danish Hospital for Rheumatic Diseases, University Hospital of Southern Denmark, Sønderborg, Denmark; 2 Department of Regional Health Research, University of Southern Denmark, Odense, Denmark; 3 Department of Rheumatology, University Hospital Lausanne (CHUV), University of Lausanne, Switzerland

## Abstract

**Objectives:**

Large language models (LLMs) are increasingly used by clinicians, yet most clinical interactions remain disposable: a question asked, an answer received, and the reasoning discarded. Just as the stethoscope required physicians to learn a skill before it became clinically useful, LLMs demand a learned technique, prompting, to unlock their potential. We present a practical framework for progressing from passive artificial intelligence (AI) use to reusable, structured clinical reasoning prompts.

**Methods:**

We propose a 5-level hierarchy of clinician-to-AI interaction, progressing from search (level 1), through conversational prompting (level 2), persistent prompting (level 3), and document-grounded project workspaces (level 4), to structured clinical reasoning prompts, termed skills, versioned, auditable, and iteratively improvable (level 5). The framework is illustrated with a proof-of-concept skill: a multidisciplinary diagnostic deliberation prompt for complex clinical cases, deployed as an open-access web application.

**Results:**

Each level is intended to add structure, reproducibility, and clinical value, though this has not been formally evaluated. Skills prompt clinical reasoning into reusable protocols with defined inputs, logic gates, verification safeguards, and tiered outputs. The case example demonstrates that clinicians can author such prompts in natural language without software engineering expertise, applying principles of version control and failure-driven iteration familiar from clinical research.

**Conclusions:**

Prompting is an acquired clinical skill, not an innate ability. A shared framework and vocabulary for structured AI interaction can help clinicians, educators, and policymakers move from disposable AI use towards more structured, auditable approaches to AI-assisted reasoning that support, but do not replace, clinical judgement.

## INTRODUCTION

Artificial intelligence (AI) in medicine spans a broad spectrum. Task-specific models can detect joint swelling from hand photographs [1] or classify sacroiliitis on pelvic radiographs [2]; domain-trained models process structured patient records to predict future disease onset [3,4]. These tools receive clinical data and return a defined output: a classification, a probability, or a risk score, but they do not generate new information, and they require no prompting from the clinician, as the task is fixed during model training. Models fine-tuned on medical data can assist with tasks such as differential diagnosis or treatment recommendation but are not accessible to the average clinician through consumer-facing platforms.

In contrast, general-purpose large language models (LLMs), tools increasingly available through platforms like ChatGPT or Claude, can generate new reasoning, explanations, and clinical narratives in response to open-ended questions. They do, however, carry no built-in medical specialisation; their clinical utility depends entirely on how they are prompted, making the quality of the prompt a critical methodological factor.

Within 3 years of their emergence, LLMs were shown to encode clinical knowledge at a level comparable with medical professionals on standardised benchmarks [5], prompting a wave of research into their potential across healthcare [6]. In rheumatology, where clinicians routinely synthesise clinical findings, blood results, imaging, and pattern recognition to navigate multisystem diseases and overlapping phenotypes, the possibilities are particularly compelling [7,8].

Many physicians already interact with AI-based tools regularly. Search engines, clinical reference platforms, and conversational AI assistants have become part of daily workflows across specialities [9]. Yet the vast majority of these interactions are disposable: a question is asked, an answer is received, and the reasoning is never saved. Each encounter starts from scratch. As medical knowledge grows at a pace that outstrips traditional formats for learning and retrieval [9], this pattern of 1-shot, nonreproducible AI use represents a significant missed opportunity.

The field of prompt engineering has matured rapidly. Early work demonstrated that LLMs could perform tasks they had never been explicitly taught, simply by being given examples in plain language [10]. Chain-of-thought prompting, where the model reasons step-by-step, measurably improves output quality [11], and reasoning models have since demonstrated accuracy exceeding physician benchmarks in diagnostic and management scenarios [12]. A scoping review identified over 100 studies applying prompt engineering to medical tasks [13]. However, this literature remains largely technical, focused on model performance rather than on teaching clinicians how to prompt.

**Figure 1:**
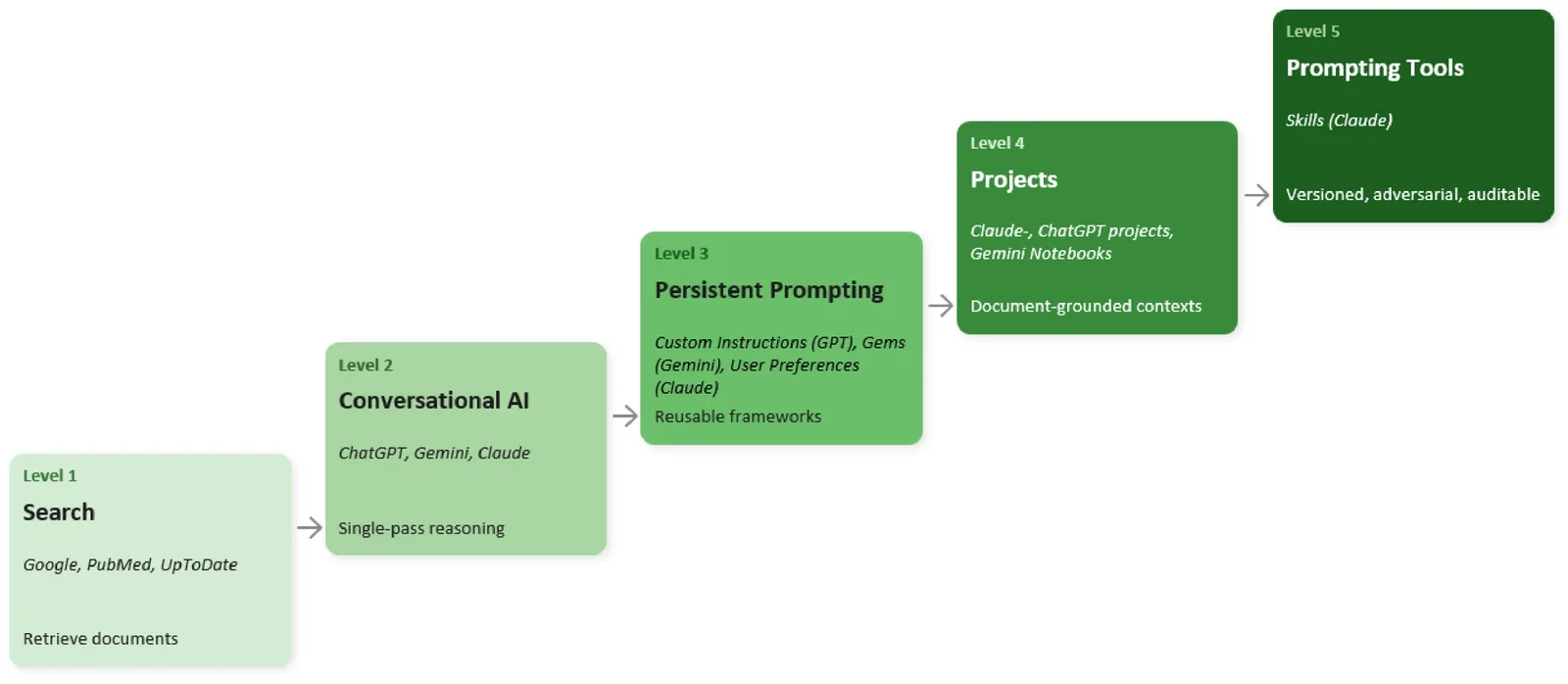
From search to skills: 5 levels of clinical AI interaction. A conceptual framework illustrating the progressive levels of clinician-to-artificial intelligence (AI) interaction, ordered by increasing structure, reproducibility, and clinical value. GPT, Generative Pretrained Transformer.

What is missing is a practical framework that takes clinicians from passive, disposable search to the systematic construction of reusable AI reasoning tools—or, borrowing from software engineering, which helps clinicians stop repeating themselves [14]. This position paper provides that framework. We describe a 5-level progression (Fig 1) from simple search to structured prompts that can be built once, improved iteratively, and shared across specialities. As a position paper, this article’s primary contribution is the conceptual framework itself. It is illustrated, but not validated, by a single proof-of-concept example: a structured reasoning skill, demonstrated through a companion web application. The example is included solely to make level 5 more concrete; neither the skill nor the application is presented as a finished, tested, or generalisable tool.

## LEVEL 1—THE SEARCH ERA: GOOGLE et al AS IMPLICIT CLINICAL REASONING

When physicians encounter a clinical question, most reach for familiar tools: Google, PubMed, UpToDate, or speciality guidelines. Survey data show that UpToDate and general search engines are the most commonly used point-of-care resources among both specialists and primary care providers, with the majority of clinicians reporting no formal training in advanced search strategies [15]. These tools work well for isolated, factual questions such as a drug dose, a classification criterion, or a guideline recommendation. PubMed alone indexes over 36 million articles, yet a typical query returns hundreds of results, and fewer than 20% of retrieved articles are directly relevant to the user’s need [16].

The limitation becomes apparent with complex clinical presentations. Consider a patient with Raynaud’s phenomenon, interstitial lung disease, and a positive anti-nuclear antibodies-titre. A Google search can retrieve information about each finding individually, but it cannot weigh them against each other, reason about the probability space of overlapping diagnoses, or carry context from 1 query to the next. Clinical reasoning at this level requires synthesis across domains—integrating serology, imaging, and pattern recognition simultaneously—a process that expert clinicians describe as iterative, contextual, and dependent on prior experience [17]. Search engines, by design, retrieve documents; they do not reason across them [16]. This is particularly limiting in fields such as rheumatology, where multisystem diseases are the rule rather than the exception [8].

Yet every search query contains an implicit prompt: a clinical question shaped by the physician’s reasoning, stripped down to a few keywords. Recognising this—making the implicit explicit—is the first step towards more effective use of AI, and the foundation of the levels that follow.

## LEVEL 2—THE PROMPTING ERA: CONVERSATIONS WITH LLMS

The release of conversational AI tools such as ChatGPT, Claude, and Gemini marked a fundamental shift in how physicians can interact with medical information. Rather than entering keywords into a search engine, clinicians could now pose clinical questions in natural language and receive synthesised, narrative responses [18]. Early surveys found that more than half of physicians and medical students who trialled these tools rated them positively for clinical use [18], and in rheumatology specifically, ChatGPT-4 was shown to achieve diagnostic accuracy comparable to that of residents in standardised case vignettes [19].

**Figure 2:**
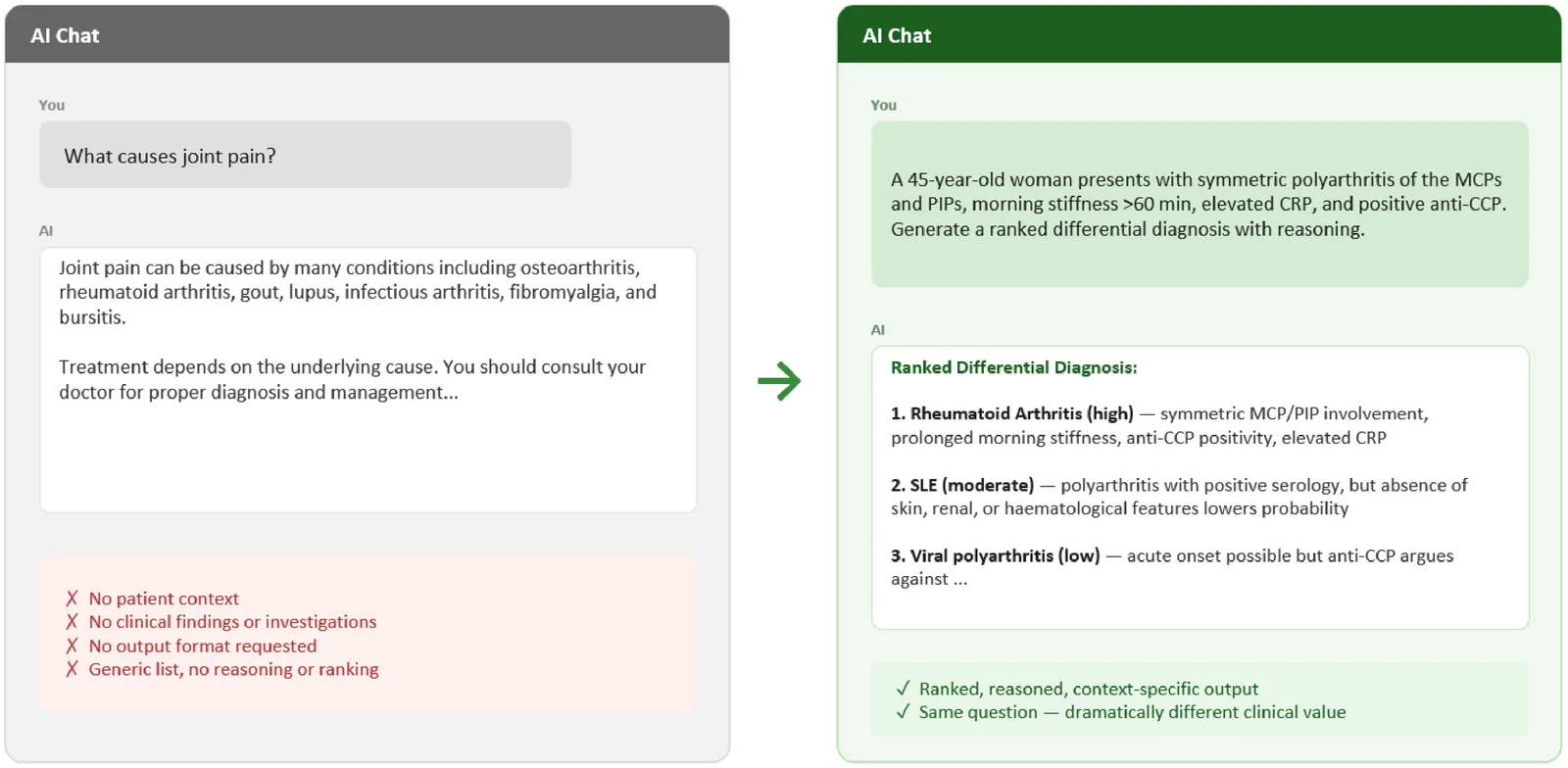
From vague query to structured prompt. Impact of prompt specificity on clinical artificial intelligence (AI) output quality. Side-by-side comparison of a vague query (left) and a structured clinical prompt (right) submitted to a conversational AI model. anti-CCP, anticyclic citrullinated peptide antibody; CRP, C-reactive protein; MCP, metacarpophalangeal joint; PIP, proximal interphalangeal joint; SLE, systemic lupus erythematosus.

However, output quality is probabilistic and depends heavily on input quality. Compare 2 prompts about the same clinical problem: ‘What causes joint pain?’ will return a generic textbook list. In contrast, ‘A 45-year-old woman presents with symmetric polyarthritis of the metacarpophalangeal joints and proximal interphalangeal joints, morning stiffness exceeding 60 minutes, elevated C-reactive protein, and positive anticyclic citrullinated peptide – generate a ranked differential diagnosis with reasoning’ produces a structured, context-specific analysis (Fig 2). Research on prompt engineering has consistently demonstrated that providing step-by-step instructions, clinical context, and explicit output requirements leads to measurably better model performance [11,13].

Yet even a well-crafted prompt remains a single-use event. When conversational AI tools first became widely available in 2023, models had no memory of previous conversations, so identical reasoning had to be rebuilt from scratch with each new session. Outputs were not reproducible—the same prompt could yield different responses on different occasions—and there were no built-in safeguards against hallucination or adversarial checking of the model’s own conclusions [6]. By 2025, all major platforms had introduced persistent memory and user preferences, partially addressing the continuity problem. Yet even with memory, the underlying limitation remains; single-turn prompting offers no mechanism to enforce structured reasoning, ensure critical diagnoses are not overlooked, or guarantee consistent output quality across clinicians. The question then becomes: what if clinical reasoning could be captured once and reused?

## LEVELS 3 AND 4—FROM PERSISTENT INSTRUCTIONS TO STRUCTURED CLINICAL CONTEXTS

A key limitation of single-use prompting is that clinicians must rebuild their reasoning framework from scratch with every new conversation—a process that has been described as ‘prompt fatigue’ [20]. Current LLM platforms address this at 2 distinct tiers of persistence.

The first tier, level 3, allows users to store reusable instructions that apply across conversations. ChatGPT offers Custom Instructions and user-built Generative Pretrained Transformers, enabling users to define reusable preferences, workflows, and response styles that persist across conversations. Google’s Gemini offers a comparable feature called Gems, packaging a prompt with a name and description so it can be reused and shared. Though Gems also support limited file uploads, their primary function at level 3 is persistent instruction packaging. Claude offers User Preferences—persistent style, tone, and formatting instructions that apply across all conversations—though it enters the persistence spectrum more fully at the project level described below. In a clinical context, a persistent instruction set might direct the model to always consider inflammatory, infectious, malignant, and metabolic aetiologies when evaluating a case—ensuring a minimum standard of diagnostic breadth. Research has confirmed that even minor differences in prompt structure can substantially alter output quality [20], and that structured clinical reasoning templates measurably improve diagnostic accuracy compared with unstructured queries [21]. These features solve the consistency problem: the same reasoning framework applies every time, without requiring the clinician to retype it. In clinical terms, this tier is analogous to verbal instructions given to a trainee—it sets expectations but does not control how the work is carried out. The model still decides the output format, but there is no mechanism for multiperspective reasoning, adversarial verification, or version control.

The second tier, level 4, moves beyond stored instructions to dedicated workspaces that combine persistent instructions with uploadable knowledge files. Claude Projects, ChatGPT Projects, and Google’s Gemini Notebooks all offer this functionality: clinicians can upload clinical guidelines, classification criteria, or formularies that the model treats as reference material across multiple conversations within that workspace. This is analogous to handing a trainee a written protocol with reference materials attached—a meaningful step up in structure and grounding.

Yet even a well-constructed project workspace remains a set of preferences rather than an enforceable protocol. In practice, project workspaces also carry computational overhead—the model processes all uploaded context with each query, which constrains conversation length and can limit the depth of reasoning in complex cases. There is no mechanism to guarantee that the model follows specific logic gates, produces outputs in a defined structure, or checks its own reasoning against a mandatory verification step. The model may comply—or it may not, and the user has no reliable way to audit this.

Several platforms are beginning to offer tools that address these limitations. Claude supports structured instruction files called skills, which can include explicit decision logic, tiered output formats, and version control. Critically, once built, a skill can be deployed and used within Claude’s standard chat interface—no terminal required. Developer-facing tools such as Gemini command-line interface are beginning to offer structured instruction files and enforcement hooks with broadly comparable capabilities, but because they require terminal-based workflows and software engineering literacy and cannot currently be invoked natively within an ordinary chat interface, they are excluded from the clinical interaction hierarchy in Figure 1. At present, Claude is therefore the only platform on which a skill in the sense defined here can be both authored and invoked within routine clinical use. These tools nonetheless represent a qualitatively different level of AI interaction: prompting reasoning protocols that are reusable, auditable, and iteratively improvable. To our knowledge, the concept of a reusable, structured AI reasoning tool has not been articulated or demonstrated for clinical audiences. The following sections aim to bridge that gap, using a worked example built with Claude’s skill architecture.

## LEVEL 5—STRUCTURED PROMPTING AS REUSABLE CLINICAL TOOLS

In software engineering, the don't repeat yourself principle holds that every piece of knowledge should have a single, authoritative representation within a system [14]. Applied to clinical AI, this means that if a physician has developed a reliable method for working through a complex diagnostic problem, which method should be encoded once and reused—not reconstructed from memory with every new conversation. In this article, we use the term skill to describe the result: a structured, versioned prompt with defined inputs, outputs, logic gates, guardrails, and quality controls that governs how an AI model reasons through a clinical task (Table 1).

**Table 1:** Maturity levels of AI tools for clinical use

Feature	Level 1 Search (Google, PubMed, and UpToDate)	Level 2 Conversational AI (ChatGPT, Gemini, and Claude)	Level 3 Persistent prompting (Custom Instructions [GPT], Gems [Gemini], and User Preferences [Claude])	Level 4 Projects (Claude Projects, ChatGPT Projects, and Gemini Notebooks)	Level 5 Prompting tools (Skills [Claude])
Reasoning	None	Single-pass	Single-pass, guided	Single-pass, document-grounded	Multiperspective and adversarial
Reproducibility	Low	Low	Moderate	Moderate	High
Memory	None	Session only	Persistent instructions	Persistent instructions + knowledge files	Versioned, transferable, and auditable
Output control	None	Prompt-dependent	Template-guided	Template-guided, document-referenced	Structured tiers
Shareability	URL sharing	Copy-paste	Exportable templates	Workspace-bound	Versioned and peer-reviewable
Auditability	None	Chat logs	Chat logs	Chat logs	Version history and logic documented
Clinical guardrails	None	None	User-defined	User-defined	Built-in verification gates
Barrier to entry	None	Low	Low–Moderate	Moderate	Moderate (conceptual)

AI, artificial intelligence; GPT, generative pretrained transformer.

Comparing features across 5 levels of increasing sophistication, from basic search to structured prompting tools (skills). Each level is illustrated with current platform examples. Features are compared across reasoning depth, reproducibility, memory persistence, output control, shareability, auditability, clinical guardrails, and barrier to entry.

Defined generally, level 5 denotes a structured prompt with 4 properties: it can be invoked directly by name within an ordinary chat interface; it enforces a defined reasoning procedure rather than merely suggesting 1, through explicit logic gates and mandatory verification steps; it is versioned, so that a specific iteration can be identified and cited; and it is auditable, so that its reasoning steps can be inspected. A tool meeting these 4 criteria constitutes a level 5 instrument regardless of the platform on which it is built.

The principle that structuring a process can improve the consistency of an outcome, independent of who carries it out, is well established in clinical care: a Cochrane review of 58 studies found that structured, multidisciplinary clinical pathways reduced in-hospital complications and length of stay, and increased adherence to recommended practice, compared with unstructured care [22]. A skill applies a structurally analogous principle to AI-assisted reasoning: rather than relying on an unstructured prompt formulated anew with each use, it fixes the inputs, logic gates, and verification steps in advance, so that the same reasoning process is applied consistently regardless of who invokes it. This parallel is structural, not evidentiary, as skills of this kind have not been subject to formal evaluation of their effect on clinical outcomes. The principle of building a repeatable clinical task into a reusable, versioned tool is not unique to language models. In rheumatology, DETECTRA—a convolutional neural network trained to detect joint swelling from dorsal hand photographs—demonstrates the same design philosophy: identify a consistent clinical task, build the method, version it, and validate it for reliable deployment [1]. A clinical reasoning skill shares this philosophy but differs fundamentally in mechanism. DETECTRA is a supervised learning model trained to answer 1 specific diagnostic question; a general-purpose language model guided by a structured prompt can generate new reasoning and respond to whatever clinical question is posed. The technologies are distinct, but the commitment to turning clinical expertise into reliable, reusable tools is the same.

It is worth situating structured prompting tools relative to established clinical decision support systems (CDSSs). Traditional CDSSs are rule-based alerts, order-set logic, drug-interaction checkers, and guideline-embedded prompts; this makes them deterministic, narrowly scoped, and typically validated for a specific task, and their reliability derives from the fact that they do not reason freely. A skill is different, as it applies a general-purpose reasoning model to an open-ended problem, generating novel synthesis rather than firing a predefined rule. The 2 are therefore complementary rather than competing. Structured prompting is thus best understood as a new and complementary category of reasoning aid, not as a replacement for validated decision support tools. Although a skill as described here governs the reasoning of a single general-purpose LLM, emerging agentic architectures allow such skills to orchestrate workflows that include the AI retrieving information from external sources and invoking specialised tools, an evolution beyond the scope of this article.

Currently, the platforms that support this level of structured prompting are limited and developer facing. Importantly, the barrier to building a skill is primarily conceptual rather than technical: the prompt is written in natural language, not code, and a clinician who can articulate the steps of their own diagnostic reasoning, which categories to consider, which findings to weigh, which diagnoses must not be missed, already possesses the core clinical knowledge required. This lowered technical barrier should not, however, be mistaken for ease of safe implementation. Articulating one’s reasoning is necessary but not sufficient: building a tool that reliably incorporates logic gates, verification steps, and clinical safeguards is a demanding, iterative process, as the 12-version development history of the example skill in section 7 illustrates. Ensuring such tools are safe for clinical use requires structured testing, institutional governance, and peer review, the same standards applied to any other clinical instrument. The framework lowers the barrier to entry; it does not remove the need for rigour. This is especially relevant in rheumatology, where patients routinely present with overlapping multisystem phenotypes that demand structured differential reasoning across categories—autoimmune, autoinflammatory, infectious, malignant, metabolic, and mechanical. This is exactly the kind of reasoning that benefits from structured prompting: complex enough to require systematic structure, yet consistent enough in its logic to be prompted once and applied repeatedly.

A further consideration is what a skill contributes when frontier general-purpose models already perform well on clinical tasks. Recent independent evaluation has demonstrated that frontier LLMs can outperform specialised, commercially tuned clinical AI products across medical knowledge, clinician alignment, and real-world clinical queries [23]. That evaluation, however, compared general-purpose models with domain-specific commercial products, not structured with unstructured prompting of the same model. A skill does not compete with a frontier model but operates upon 1. Its intended contribution is therefore not improved diagnostic accuracy, but greater consistency, auditability, reproducibility, educational value, and workflow efficiency in how the underlying model is applied. A skill enforces the application of identical reasoning steps, verification gates, and safety checks on every occasion, and yields a versioned, inspectable record of how a given conclusion was reached. In doing so, it is intended to support the clinician’s own reasoning and reduce the likelihood that a relevant diagnostic category is overlooked; any consequent improvement in decision quality would derive from a more disciplined reasoning process rather than from a validated increase in the accuracy of the tool itself. The following section presents a worked example of such a skill, developed for complex, multisystem diagnostic reasoning.

## CASE EXAMPLE—A SKILL: FROM CONCEPT TO DEMONSTRATION

The skill described in this section is presented as a proof-of-concept. It is not a validated CDSS and should not be used as a substitute for clinical judgement. Its purpose is to make the abstract concept of a ‘skill’ concrete, reproducible, and testable.

### Skill overview and architecture

**Figure 3:**
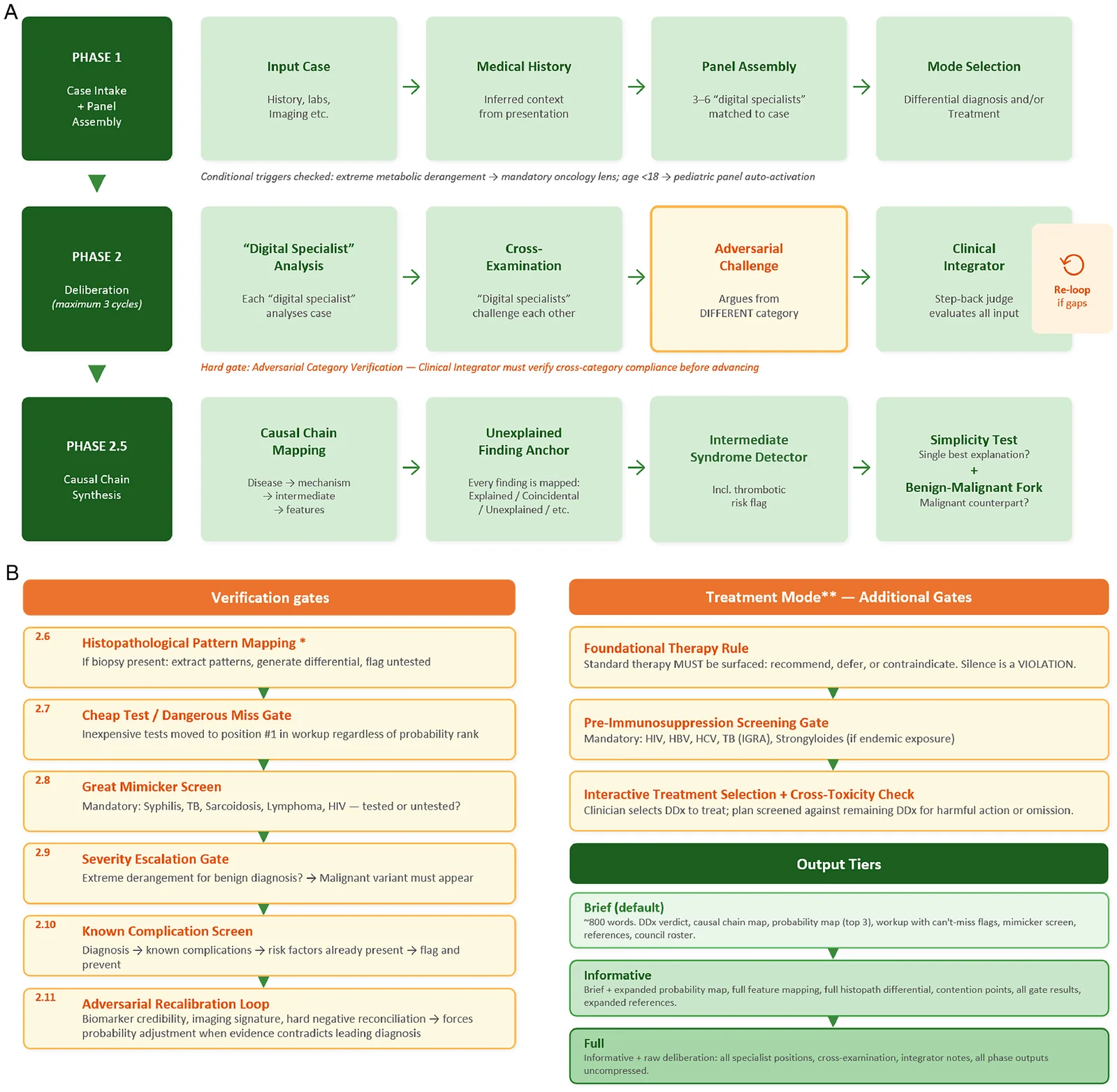
(A) Structured deliberation pipeline of skill—illustrating case intake and panel assembly (phase 1), adversarial deliberation (phase 2), and causal chain synthesis (phase 2.5). Green boxes represent sequential processing steps. Orange boxes indicate adversarial or challenge mechanisms. Orange text denotes hard gates—conditional checkpoints that must be satisfied before the pipeline advances. The reloop arrow in phase 2 indicates that deliberation may cycle up to 3 times if the digital Clinical Integrator identifies unresolved gaps. (B) Verification gates and output structure of the skill—illustrating sequential safety checks after deliberation (steps 2.6-2.11), additional gates in treatment mode, and tiered output depth (Brief, Informative, and Full). The right column shows additional gates activated in treatment mode, and the 3 output tiers (Brief, Informative, Full) with increasing depth of detail. Orange boxes represent verification and treatment-mode gates. Green boxes represent output tiers. * A conditional gate that only activates when relevant data are present (eg, biopsy results). ** Treatment mode, activated when the clinician requests treatment guidance in addition to differential diagnosis. DDx, differential diagnosis; HBV, hepatitis B virus; HCV, hepatitis C virus; IGRA, interferon-gamma release assay; TB, tuberculosis.

*The Medical Council* is a skill that simulates a multidisciplinary team (MDT) deliberation for complex clinical cases. When given a case, it assembles a virtual panel of 3 to 6 specialists matched to the clinical presentation, runs structured deliberation cycles in which each specialist analyses the case and is then challenged from the perspective of a competing diagnostic category, and synthesises the output into a ranked differential diagnosis with explicit reasoning. The architecture—shown in Figure 3A,B—includes a series of verification gates designed to catch common reasoning failures: a screen for frequently missed diagnoses (syphilis, tuberculosis, sarcoidosis, lymphoma, and HIV), a gate that prioritises cheap and readily available tests before expensive workup, a severity check that forces consideration of malignant variants when findings are too extreme for a benign diagnosis, and a complication screen that flags known downstream risks of the leading diagnosis. The skill handles both adults and children, operates in diagnostic and treatment modes, and produces output at 3 levels of detail. The skill is now in its twelfth version (v12), with each iteration driven by failure analysis against published clinical cases; when the skill gets a case wrong, the architecture is revised to prevent that class of error from recurring. This iterative process drew on an initial set of 8 cases presented at the European Alliance of Associations for Rheumatology (EULAR) 2026 hands-on workshop ‘Clinical Case Solving in Rheumatology – With and Without AI Support’, followed by approximately 20 published case reports from the *New England Journal of Medicine Case Challenges*. For example, an early version failed to synthesise a diagnosis of kwashiorkor secondary to dysphagia despite correctly identifying each contributing finding individually, prompting the addition of a dedicated intermediate-syndrome detection step; a later version correctly identified hyperaldosteronism but defaulted to a benign aetiology, missing an underlying adrenocortical carcinoma, prompting a dedicated malignancy-exclusion gate.

The choice of underlying model to use with the skill was also examined. A structured comparison of several model configurations, spanning different base models with and without extended (‘adaptive’) reasoning, was conducted across a small set of clinical vignettes, scored against a multidimensional quality rubric and a must-flag safety-coverage check. Output quality was largely insensitive to the choice of base model, and enabling extended reasoning produced a modest quality gain at a substantial cost in output length and latency. Model configuration therefore represents a tunable trade-off between quality, cost, and speed rather than a single optimum, a balance that can be re-evaluated for any given version and deployment context. The evaluation also surfaced a gap common to all configurations; none reliably produced explicit weight-based paediatric dosing, identifying this as a structural limitation of the skill itself rather than of any specific model, and directly motivating the paediatric dosing revision introduced in a later version.

Subsequent iterations continued to address reasoning gaps surfaced by individual cases. For example, the skill’s handling of contradictory evidence was strengthened: in cases where the correct diagnosis was ultimately missed despite the presence of incompatible findings, an adversarial recalibration step was added, requiring the panel to formally score evidence that argues against its own leading diagnosis and to re-rank when a finding is genuinely incompatible.

**Figure 4:**
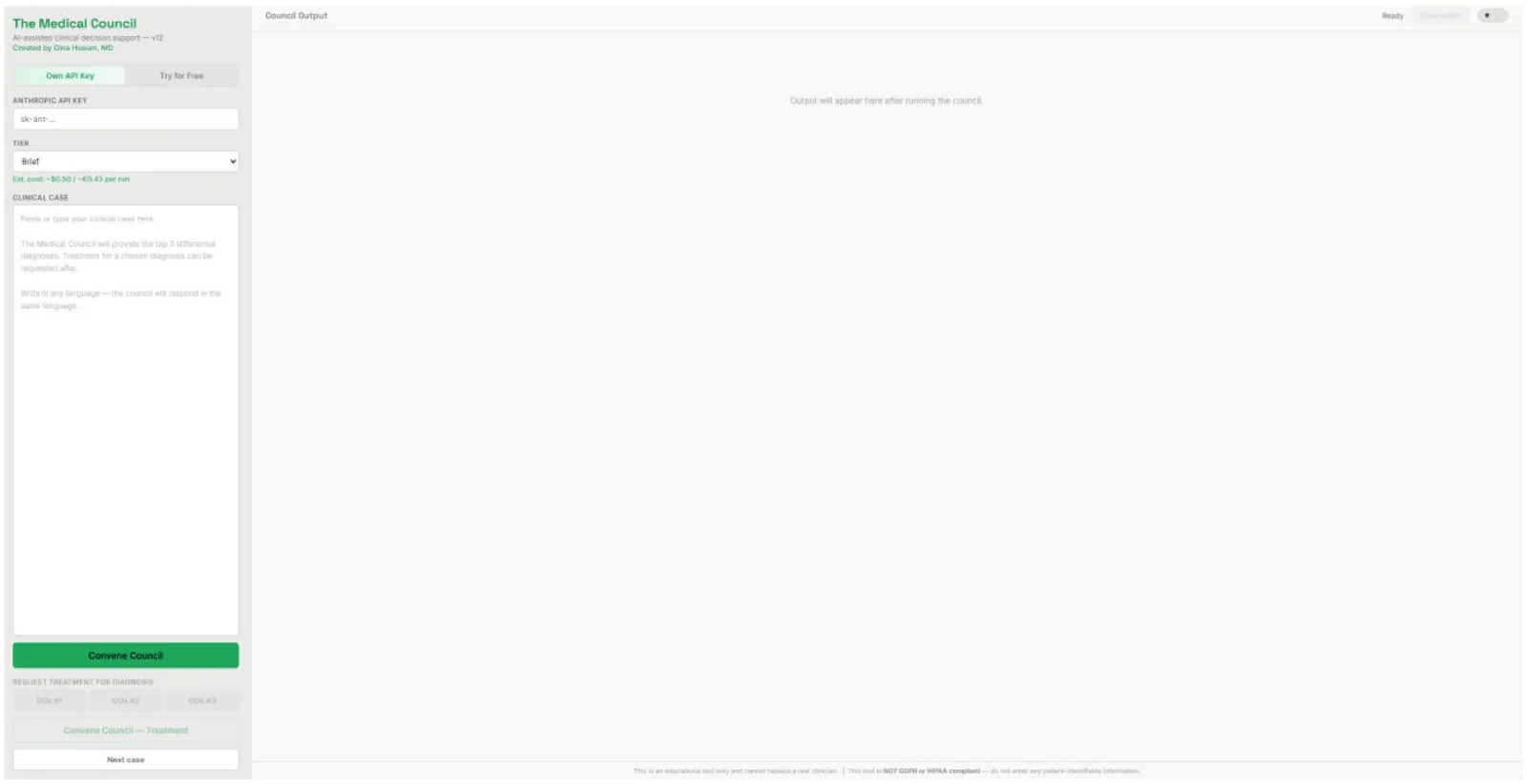
Proof-of-concept web application for the skill. Web interface of the skill at www.dxmypt.com—a proof-of-concept application allowing clinicians to run the skill directly in-browser. Users paste a clinical case, select an output tier (Brief, Informative, or Full), and press ‘Convene Council’ to generate a ranked differential diagnosis. Treatment plans can be requested for selected diagnoses via ‘Convene Council—Treatment’. The skill responds in the language of the input case. Left panel: case input with tier selection and API key entry. Right panel: council output display. The application offers 3 free runs per reader for the first to test it; continued use requires the user’s own Anthropic API key. API, application programming interface.

To supplement this description, a companion web application (Fig 4) was developed, allowing for interacting with the skill. Interface and access details are provided in the Figure 4 legend.

### Clinical rationale

No single clinician holds every differential diagnosis across every speciality; this is precisely why MDT meetings exist, bringing together perspectives that no individual can maintain alone. In practice, however, an MDT is not available for every complex case, at every hour, or in every setting where specialist access is limited. A structured reasoning tool of this kind is intended to help address that gap, not as a replacement for colleagues, but as a structured thinking partner that prompts the clinician to consider diagnoses that might not appear on an initial mental shortlist. In principle, a clinician assessing a patient with overlapping autoimmune, infectious, and malignant features could be supported by a tool designed not to collapse those categories into a single explanation before the alternatives have been actively considered.

The intent is cognitive support, not automation. A skill of this type does not make decisions: it is designed to present a structured differential, highlight commonly missed diagnoses, and challenge the leading hypothesis, before returning the reasoning to the clinician, with whom responsibility remains.

### Implications and transferability

This case example is not intended to suggest that a single skill can replace clinical expertise. Rather, it illustrates that the principles described in this article—structured reasoning, version control, and iterative improvement through failure analysis—can be applied to clinical AI tools by clinicians themselves, without software engineering expertise. The architecture is transferable: a cardiologist, neurologist, or endocrinologist could build an equivalent tool using the same structural principles, tailored to the diagnostic patterns of their own speciality. The example skill is the proof-of-concept; the method is the message.

## LIMITATIONS, RISKS, AND RESPONSIBLE USE

The framework described in this position paper is subject to important limitations that must be acknowledged openly.

First, all current LLMs are susceptible to hallucination—generating outputs that are plausible in tone but factually incorrect or entirely fabricated [6]. Skills can be designed to mitigate this risk through structured guardrails, mandatory verification gates, and adversarial challenge mechanisms; however, the extent of this mitigation has not been formally measured, and such mechanisms do not eliminate the risk of hallucination. A skill’s output remains a probabilistic generation, not a deterministic calculation, and clinical judgement is irreplaceable at every stage of interpretation.

Second, clinician-authored skills are reasoning aids, not validated CDSSs. They have not undergone the regulatory evaluation required of Conformité Européenne-marked or Food and Drug Administration-cleared medical devices, and they should not be treated as such. The distinction between a physician-authored reasoning tool and a regulated medical device is an area of active regulatory development, particularly under the EU AI Act and Medical Device Regulation, where the classification of physician-authored reasoning tools remains an area of active development [24].

A further limitation concerns platform dependence. The 5-level framework and its underlying principles, structured reasoning, version control, and iterative improvement through failure analysis, are platform-general. What is currently platform-specific is the ability to deploy and invoke that file as an enforceable skill within an ordinary clinical chat interface, calling it by name, with adhered-to logic gates and version control. At the time of writing, this is realised in a clinician-usable form only through 1 commercial provider (Anthropic’s Claude); on other consumer platforms, a comparable instruction file must be reattached to each conversation or embedded within a project or custom assistant, without the same named invocation or enforcement. This is why level 5, as defined here, is presently occupied by a single platform. Readers should therefore distinguish the framework and principles, which are general, from the current means of realising level 5, which is not; as enforceable, natively invocable structured prompting becomes available elsewhere, the same framework is intended to apply without modification.

Third, data privacy must be treated as a non-negotiable constraint. As a practical rule, identifiable patient-level information should never be entered into a commercial AI platform; cases should be deidentified before entry, and use should comply with institutional information-governance approval and any applicable data-processing agreements. Current commercial LLMs store conversational data on external servers, and the risk of inadvertent disclosure of protected health information remains a serious concern, particularly under the European General Data Protection Regulation [25]. Clinicians using these tools must ensure compliance with local data governance policies.

Fourth, there is the risk of overtrust. Structured, authoritative-looking output can create a false sense of confidence, particularly when the output includes references, probability estimates, and specialist panel framing. Research on automation bias in clinical AI has shown that physicians can be influenced by AI-generated recommendations even when those recommendations are incorrect, and that this effect is amplified when the output appears systematic and well reasoned [26]. Ultimately, accountability for any clinical decision rests with the clinician, not the tool: a skill’s output is advisory only, and the responsibility for verifying, accepting, or rejecting it cannot be delegated to the model.

A skill should be used to broaden and structure clinical reasoning, to widen a differential or surface an overlooked possibility, not to reach or confirm a diagnosis autonomously. Its output should always be independently verified against primary sources and the clinician’s own judgement, never treated as a final recommendation, and treatment-related outputs require independent confirmation of dosing and contraindications against an authoritative reference. These boundaries apply to all current clinician-authored reasoning tools, irrespective of platform.

## CONCLUSION AND FUTURE DIRECTIONS

The progression from search to skills is not about replacing clinical reasoning—it is about supporting it. In a busy clinical day, experienced physicians risk overlooking rare differentials, forgetting to consider a diagnostic category, or missing a pattern that might have been caught with a second perspective. A well-built skill may help address this: by structurally prompting consideration of a broad range of possibilities, it can serve as an aid that reduces the likelihood of an overlooked category or missed pattern, supporting the consistent application of the clinician’s own knowledge under time pressure. Whether it reliably achieves this in practice remains to be demonstrated. This position paper has described a 5-level framework that takes physicians from disposable, 1-shot searches to the construction of reusable, versioned, and auditable reasoning tools that can serve that role. The framework is platform-general, the conceptual barrier is surmountable, and the tools to begin building exist now. What has been missing is the vocabulary and the worked example to make the concept accessible to clinicians. This paper provides both. The 5-level framework itself offers a shared vocabulary for researchers, educators, and policymakers to describe and evaluate how clinicians interact with AI—from passive retrieval to active prompt-building.

Rheumatology is well suited to contribute to this area. The diagnostic complexity of multisystem autoimmune and autoinflammatory diseases demands exactly the kind of structured, multiperspective reasoning that codified tools can provide. The same rigour that the speciality brings to classification criteria, treat-to-target protocols, and clinical trial design can and should be applied to the development of AI reasoning tools. We encourage clinicians to approach skill development with the same methodological discipline as any other research output, building, sharing, and publishing tools whose performance has been examined rather than assumed.

Several developments are likely to accelerate this trajectory. Integration with electronic health records and real-time patient data would remove the current reliance on manual input, allowing skills to operate on structured clinical context directly. The convergence of reasoning tools with digital biomarkers—for example, a workflow in which DETECTRA’s joint swelling data feeds into a diagnostic reasoning skill alongside laboratory values and imaging [1]—represents a natural next step towards integrated, AI-augmented clinical decision-making. Agentic workflows, in which skills can autonomously search the literature, cross-reference guidelines, and interrogate drug-interaction databases, are already technically feasible and will further expand what clinician-authored prompts can achieve.

The collaborative potential is significant. Because skills are written in natural language and are platform-transferable, they can be shared, peer-reviewed, and improved collectively—much like clinical guidelines. The infrastructure for this is already emerging. The Digital Rheumatology Network, now in its sixth year, has established itself as an international community connecting clinicians, researchers, and technology developers around digital innovation in rheumatology [27]. Platforms such as this, alongside professional societies and working groups within EULAR, offer a natural home for curated, peer-reviewed skill libraries—shared repositories where clinician-built reasoning tools can be versioned, validated, and refined collectively, enabling the standardisation of AI-assisted clinical reasoning across departments, institutions, and borders.

## CRediT authorship contribution statement

**Dina Husum:** Writing – review & editing, Writing – original draft, Visualization, Project administration, Methodology, Conceptualization. **Thomas Hügle:** Writing – review & editing, Supervision, Methodology, Conceptualization.

## Competing interests

All authors declare they have no competing interests.
